# Insights into rare diseases from social media surveys

**DOI:** 10.1186/s13023-016-0532-x

**Published:** 2016-11-09

**Authors:** William Davies

**Affiliations:** 1 Medical Research Council Centre for Neuropsychiatric Genetics and Genomics and Division of Psychological Medicine and Clinical Neurosciences, School of Medicine Cardiff University, Cardiff, UK; 2 School of Psychology, Cardiff University, Tower Building, 70, Park Place, Cardiff, CF10 3AT UK; 3 Neuroscience and Mental Health Research Institute, Cardiff University, Cardiff, UK

**Keywords:** Facebook, Response bias, Social media, Survey, Twitter, X-linked ichthyosis

## Abstract

The internet, and social media platforms, are increasingly being used by substantial sectors of the worldwide population. By engaging effectively with online and social media, scientists and clinicians can obtain unprecedented access to relatively large cohorts of individuals with rare diseases, as well as their relatives, carers and professionals involved in their healthcare. Online surveys of these stakeholder groups may provide important new insights into rare conditions and their management relatively quickly and easily, with the possibility of rapid translation into healthcare interventions and policy. Here, I describe our recent positive experience with the online survey approach to a rare disease (X-linked ichthyosis), and review its advantages and limitations.

## The rise of the internet and social media

More than 40 % of the world’s population now has regular access to the internet, a figure which is increasing rapidly as a consequence of cultural factors and the more widespread availability of high-speed broadband technology [1]. A high proportion of internet users engage with social media, online platforms via which individuals and groups from across the world may correspond with one another; the two best known social networks, Facebook (https://www.facebook.com) and Twitter (https://twitter.com), currently have over 1.7 billion and 310 million active users respectively [2, 3]. These high site usage rates are likely to be due, in large part, to the fact that these services are simple to sign up to and free to use. The rise of such massive virtual communities has stimulated the foundation of a high number of social media support groups for a wide range of medical conditions ranging from the very common, to the extremely rare.

Large-scale studies have used social media sites including Facebook and other online social enterprises (e.g. Amazon Mechanical Turk) to generate insights in the field of social sciences and psychology [4, 5], but, as yet these platforms have yet to be fully exploited in the area of medical and life sciences [6]. This may be because senior investigators in the latter area are often not conversant with social media usage, and do not understand the various platforms’ capabilities and range; moreover, researchers in the field of rare disorders may have been trained using traditional clinical methods typified by low-throughput local recruitment methods and clinic-based deep-phenotyping.

Here, I argue that this traditional approach to rare diseases may be usefully supplemented through social media-focussed surveys of comparatively large cohorts of patients, their relatives and carers, and related healthcare professionals, with a view to providing quick, easy, and readily-generalizable findings of significant clinical and social relevance. Rare diseases about which additional information could be gleaned may range from those with a defined pathology but poorly-defined phenotypes (for example, some genetic copy number variant conditions), to conditions with a well-characterised phenotype but a poorly-defined aetiology (e.g. postpartum psychosis). As genome-wide analyses such as exome or whole-genome sequencing become more commonplace, it is likely that the number of patient groups on social media associated with specific pathogenic genetic mutations will increase substantially. Our experience of this complementary approach, and the advantages and limitations associated with it are described below.

## New insights into the rare disease X-linked ichthyosis (XLI): our recent experience

X-linked ichthyosis (XLI) is a rare dermatological disease, thought to affect between 1 in 3000–6000 males, and caused by deficiency for the enzyme steroid sulfatase [7]. Case studies and work in a small sample of boys with the condition had indicated that individuals with XLI may be at increased risk of developmental disorders, including Attention Deficit Hyperactivity Disorder (ADHD) and autism spectrum, and related, disorders (ASDs). We wanted to test whether these preliminary findings could be replicated, and whether behavioural traits associated with these conditions could be observed more frequently in adults with XLI than healthy controls. Hence, we developed a detailed online survey and disseminated the associated URL to relevant patient groups and charities on Facebook, on Twitter, and via relevant charity websites. Within the space of just a few months, we obtained a reasonable response rate from individuals affected by XLI or their parents (survey is still ongoing) which provided reliable new information regarding psychiatric and behavioural phenotypes in this condition [8]. During the conduct of our study and its assessment for publication, we recognised a number of advantages and limitations associated with the online survey strategy we used which we considered that it would be informative for the rare disease community to appreciate.

## Advantages of the online social media-based approach

### Use of online surveys

There are a number of low-cost platforms available for generating online surveys which require minimal background technical knowledge (e.g. via Qualtrics [9]); surveys may be custom-designed, based upon existing validated questionnaires, or a combination of the two. Survey platforms allow a broad range of questions to be asked, and permit a degree of flexibility with regard to response options (single or multiple response, or open-ended); hence, participants are free to contribute as fully, or as little, as they wish. Open-ended answers can allow patients to raise issues that may not previously have been considered or appreciated by medical professionals. Importantly, online surveys typically require very little time commitment on behalf of participants (ideally, they should not exceed 40mins to maintain high levels of participant engagement), and are returned to the researchers automatically upon completion. Through the judicious use of questionnaires, scientists and clinicians may readily obtain information pertinent to rare diseases; this might include information on the range, severity or prevalence of phenotypes associated with a particular condition, on medical and social care needs most pressing to patients and their families, or on the efficacy and potential side-effects of medications and interventions.

### Ease of administration and low social desirability bias

Provided patients are given the option of omitting questions that they feel uncomfortable answering, the online survey approach has few ethical implications; this is even truer when the data are collected anonymously. Moreover, there is no need to go through the laborious ethical processes associated with recruiting via more traditional methods involving physicians, hospitals and health boards. The lack of ethical issues associated with the online approach, coupled with the immediacy of the questionnaire completion and return, means that projects can be initiated and undertaken quickly (in our case, from development of the idea to publication of the first associated paper took less than 12 months). Such rapidity and ease of implementation means that studies of this type may be ideal for student projects where there are significant time constraints. The rapidity of implementation further means that patient and stakeholder opinions and concerns can be taken into account promptly in areas such as clinical decision-making, formulating policy or genetic counselling.

The lack of face-to-face interaction and the degree of anonymity inherent in online surveys is likely to elicit increased truthfulness from participants by reducing social desirability and central coherence biases; this may be particularly relevant for issues that are socio-culturally sensitive or which may cause embarrassment e.g. with regard to mental or sexual health or criminal behaviours [10].

### Participant engagement

The online approach means that large, geographically, socially and culturally distinct populations from around the world (or the developing/developed world at least) can be accessed. Hence, it may be possible to examine the extent to which patients from these diverse populations exhibit commonalities, and to what extent they differ; this, in turn, may highlight genetic, environmental and socio-cultural (e.g. diagnostic practices) that modulate the presentation and treatment of the disorder. Patients with rare diseases (and their relatives/carers) are often highly motivated to engage in research and particularly so where this involves a small time commitment and little effort. The online survey approach is also convenient relative to the traditional approach: participants can complete the survey at the location and time of their choosing which may be especially pertinent for patients with impaired mobility or living in remote communities for example, or for patients with competing commitments such as childcare. Theoretically, the online survey approach may reach a demographic that other research options cannot.

Engagement with enthusiastic and supportive online communities has an additional advantage in that the results of any studies can be disseminated immediately to members, as well as through more traditional avenues (e.g. academic journals, medical institutions, professional organisations), to influence clinical decision-making and policy as mentioned above.

## Limitations of the online approach

### Lack of objective participant assessment

Perhaps the most obvious limitation of an online approach relative to the more traditional approach is the reliance on information provided by the patient, which is not corroborated by objective clinical assessment or tests. For a variety of reasons, patients may unintentionally (or even intentionally) mis-report their diagnosis and clinical results, their symptoms, their medication regime, and their environmental and social circumstances; the impact of intentional mis-reporting may be attenuated by anonymity. To limit the effects of mis-reporting, or at least to be aware of its possible scope, we advise that the online survey builds in relevant control measures so far as possible; for example, in our XLI study, we could check that individuals who reported that they had been diagnosed with an ASD scored highly on questionnaire measures examining related traits.

Second, whilst traditional approaches permit in-depth longitudinal phenotyping, and a detailed picture of the (often complex) relationships between a patient’s social circumstances, various medical conditions, symptom prevalence and severity, and medication regimes to be created, an online approach, which provides a snapshot contaminated by recall or self-report bias, cannot provide extensive information on interactions between the aforementioned factors. If participants are allowed to register their interest in follow-up studies at the end of the online survey, subsequent, more intensive, studies designed to interrogate interesting preliminary findings and to illuminate mechanism may be undertaken.

#### Lack of opportunity for clarification

Although online surveys are highly accessible, they can often be fairly complex and can use technical language and terms; in contrast to studies where clinician/scientist and participant meet face-to-face, there is not an immediate opportunity for participants to clarify potential issues if required. Hence, there is the possibility that the study’s purpose, or questions within the survey, may be misunderstood.

#### Response bias

A key issue with survey-based approaches, and particularly those targeted at affected groups, is that the participants who respond are not representative of the group for one reason or another; this may result in false assumptions regarding that population. For example, the prevalence and severity of particular symptoms in patients with the condition of interest, or the frequency of adverse side effects of medication may be over-estimated if patients with that particular symptom or side-effect are more motivated to participate. In our study, we were concerned that individuals with XLI with established diagnoses of one or more behavioural disorders would be more motivated to respond than individuals with XLI but no diagnostic history of such disorders (thus inflating the apparent rate of psychological disorders in the condition). To address this issue, researchers should attempt to confirm that the demographic and clinical features of respondents approximate to those of the general patient population, and that calculated rates and symptoms of the condition under study are consistent with existing clinical and laboratory data (which may, in many cases, be rather limited).

Additionally, the data may be biased as a consequence of the survey only being accessible to computer-literate individuals who have access to the internet; moreover, as support groups and charities related to rare diseases are often based in developed countries (typically US and northern Europe), there is the possibility that the results of online respondents may not necessarily be representative of all geographical and cultural contexts. Finally, given that English is the standard language of science, non-English speaking participants may be excluded from responding. To mitigate these concerns, surveys should be advertised as widely as possible and translated where feasible, and country of residence and ethnicity of respondents (as well as any other relevant cultural measures) should be recorded. Importantly, assessment of these factors means that the extent to which measures of interest vary by geographical-cultural context can be analysed; if the measures do not vary considerably, the findings may be regarded as highly generalisable. However, it is likely that geographical-cultural context can impact significantly on some parameters: in the field of psychiatry, individuals may be diagnosed through different classification systems and criteria depending upon geographical region; moreover, inter-rater reliability in this field can vary substantially around the world.

As well as having different countries of residence and been diagnosed by variable methods, respondents to large-scale surveys may differ significantly on ‘demographic’ factors such as age, medication regime or social circumstances; this is probably less of an issue with more-focussed traditional approaches with stricter inclusion and exclusion criteria. Whilst this greater participant variability may be associated with greater variability in the response data, potentially it could be exploited as a means to examine relationships between demography and measures of interest.

#### Participant knowledge, concerns and malicious responses

Whilst responding to an online survey, participants may simultaneously use the internet to gain information on pertinent aspects of the study. This information may be reliable or not, and, in conjunction with participants’ prior knowledge and expectations, could feasibly shape their responses [11].

In traditional research studies, patients meet directly with a ‘trusted’ clinician and/or scientist and have the opportunity to build up a rapport with them. In the online world, developing such a personal relationship is more challenging, and participants may reasonably be more sceptical as to the motives of the survey particularly given the prevalence of unethical, unprofessional and pseudoscientific contributors to the internet. Our XLI survey was received extremely positively by most individuals but was criticised by some for its usefulness and relevance (‘how will understanding behaviour in patients with XLI improve their skin condition?’), and for its (non-existent) links to ‘Big Pharma’. As such, we advise that online adverts for surveys clearly and concisely indicate the expected benefits for patients, state where the research is being conducted, explain the ethical review procedures which have been undertaken, and refer to the funding bodies and any resultant conflicts of interest.

Finally, as with any publically-accessible forum, there is the remote possibility that participants could maliciously complete the survey, particularly if they disagree with its rationale or the beliefs of the authors; researchers should therefore be aware of any outlier data.

#### Obtaining suitable control groups

By targeting the survey link to patient groups and websites, by definition the primary respondents will be affected individuals. However, often these groups include close family members of affected individuals; these unaffected individuals may be regarded as the optimal control group in that they can be closely matched to patients in terms of genetic background and environmental and social exposures. In our study, we found it difficult to recruit non-affected brothers of males with XLI, and hence we compared patient survey information to the best-available previously-published gender, ethnicity and culturally-matched normative data; our approach represents a viable analytical option (albeit suboptimal) if reliable normative data already exists.

#### Consent to advertise

Online patient support groups (e.g. support groups on Facebook) are often selective about who is allowed to join and advertise; similarly, charities and academic/hospital departments understandably have to be selective about the types of study which they publicise. Hence, the surveys to be advertised may have to be given additional academic and ethical review by these external bodies. In our experience, patient group moderators, charities and medical departments are extremely happy to support such work provided it has a sound academic rationale, has been suitably ethically-reviewed and may potentially benefit patients.

## Conclusions

Online surveys advertised via social media represent a relatively new experimental method by which information from patients with rare diseases (and their relatives and carers) can be obtained in a straightforward manner, and can be used to benefit affected individuals in a short timeframe. Whilst this approach is limited in the numerous ways described above, and will never replace deep phenotyping in face-to-face studies, it can certainly provide complementary information to them. I believe that this approach may be especially useful for getting an overview of the wide range of phenotypes that affect patients (many of which may not be intuitive) and for gaining information on those issues which most impair affected individuals’ lives. These analyses should point to areas where research, funding and interventions should be directed most acutely.

## Abbreviations

ADHD: Attention Deficit Hyperactivity Disorder
ASD: Autism spectrum disorder
URL: Uniform Resource Locator
XLI: X-linked ichthyosis
